# *Spartina alterniflora* invasion reduces soil microbial diversity and weakens soil microbial inter-species relationships in coastal wetlands

**DOI:** 10.3389/fmicb.2024.1422534

**Published:** 2024-07-31

**Authors:** Tao Zhang, Bing Song, Luwen Wang, Yong Li, Yi Wang, Min Yuan

**Affiliations:** ^1^School of Resources and Environmental Engineering, Ludong University, Yantai, China; ^2^State Environmental Protection Key Laboratory of Wetland Ecology and Vegetation Restoration, Institute for Peat and Mire Research, Northeast Normal University, Changchun, China; ^3^Beijing Key Laboratory of Wetland Services and Restoration, Institute of Wetland Research, Chinese Academy of Forestry, Beijing, China; ^4^Department of Renewable Resources, University of Alberta, Edmonton, AB, Canada

**Keywords:** co-occurrence network, microbial composition, microbial functions, plant invasion, Yellow River Delta

## Abstract

Soil microorganisms play a crucial role in the plant invasion process, acting as both drivers of and responders to plant invasion. However, the effects of plant invasion on the complexity and stability of co-occurrence networks of soil microbial communities remain unclear. Here, we investigated how the invasion of *Spartina alterniflora* affected the diversity, composition, and co-occurrence networks of soil bacterial and fungal communities in the Yellow River Delta, China. Compared to the native plant (*Suaeda salsa*), *S. alterniflora* invasion decreased the α-diversity of soil bacterial communities but did not affect that of fungal communities. The β-diversity of soil bacterial and fungal communities under *S. salsa* and *S. alterniflora* habitats also differed dramatically. *S. alterniflora* invasion increased the relative abundance of the copiotrophic phylum Bacteroidota, whereas decreased the relative abundances of the oligotrophic phyla Acidobacteriota and Gemmatimonadota. Additionally, the relative abundance of Chytridiomycota, known for its role in degrading recalcitrant organic matter, increased substantially within the soil fungal community. Functional predictions revealed that *S. alterniflora* invasion increased the relative abundance of certain soil bacteria involved in carbon and nitrogen cycling, including aerobic chemoheterotrophy, nitrate reduction, and nitrate respiration. More importantly, *S. alterniflora* invasion reduced the complexity and stability of both soil bacterial and fungal community networks. The shifts in soil microbial community structure and diversity were mainly induced by soil available nutrients and soil salinity. Overall, our study highlights the profound impacts of *S. alterniflora* invasion on soil microbial communities, which could further indicate the modification of ecosystem functioning by invasive species.

## 1 Introduction

The invasion of exotic plants is one of the most important factors driving global environmental change and poses a great threat to the functioning of natural ecosystems (Carboni et al., 2018; Gioria et al., 2023). Soil microbes are both drivers of and responders to plant invasion, and they play critical roles in regulating biogeochemical cycles and maintaining ecosystem functions (Stefanowicz et al., 2016; Zhang Z. et al., 2020). Soil microbiota can promote the growth of invasive plants through various pathways, such as altering nutrient acquisition and modifying litter decomposition, thereby enhancing the environmental adaptability of invasive plants (Tamura and Tharayil, 2014; Yu et al., 2021). Meanwhile, plant invasion can alter the abundance, composition, and structure of soil microbial populations (Fried et al., 2019; Li S. et al., 2022). Nevertheless, previous research extensively focused on the aboveground ecological processes underlying plant invasion (Stanek et al., 2023). The comprehensive interaction between alien invasive plants and soil microorganisms remains unclear, and it is crucial to thoroughly understand changes in the structure and function of soil microbial communities in response to plant invasion.

*Spartina alterniflora* is one of the most widespread invasive plants in coastal zones around the world. Originally from the United States, it was introduced to China in 1979 for ecological restoration purposes (Liu et al., 2018). Since then, it has spread to most coastal areas in China, posing a severe threat to native mangrove and salt marsh ecosystems (Li H. et al., 2022). Previous studies have illustrated that *S. alterniflora* invasion can shift the accumulation and turnover of soil carbon and nitrogen pools, and modify various soil physicochemical properties (Cheng et al., 2006; Gao et al., 2017). Furthermore, *S. alterniflora* invasion can significantly affect certain specialized functional bacteria in the soil. These bacteria are involved in carbon, nitrogen, and sulfur cycling, such as methane-producing bacteria (Yuan et al., 2014), nitrogen-fixing bacteria (Huang et al., 2016), and sulfate-reducing bacteria (Zhang G. et al., 2020). Moreover, the crucial role of rhizosphere microorganisms and the presence of rare microbial communities in contributing to *S. alterniflora* invasion have been increasingly validated (Yu et al., 2022). However, at this stage, the specific effects of *S. alterniflora* invasion on the co-occurrence networks of soil bacteria and fungi are still unclear. This knowledge gap hinders a complete understanding of the interaction between *S. alterniflora* invasion and changes in soil microbial communities.

Soil microbial co-occurrence networks can reveal the intricate relationships among microbial species (Ma et al., 2020). The association, complexity, and stability within microbial networks can serve as indicators of changes in microbial structure and thus their ecological functionalities (Wagg et al., 2019; Yuan et al., 2021). For example, the importance of soil microbial co-occurrence network complexity in maintaining the relationship between soil microbial diversity and ecosystem multifunctionality has been demonstrated in different grassland ecosystems (Luo et al., 2023). A recent study found that the complexity of microbial networks indirectly drove the impact of microbial diversity on ecosystem function, and the complexity of microbial networks was a better predictor of ecosystem function than simplified diversity indices (Chen et al., 2022). However, little research has specifically addressed the effects of exotic plant invasion on the functionality and co-occurrence networks of soil microbial communities. Moreover, it remains unclear whether the co-occurrence networks of soil bacterial and fungal communities exhibit consistent responses to plant invasion, given their distinct physiological traits and environmental adaptation strategies (Barnard et al., 2013). Therefore, it is essential to simultaneously investigate the structure, function, and co-occurrence networks of soil bacterial and fungal communities for a comprehensive understanding of underground ecological processes following plant invasion.

The Yellow River Delta Wetland is the largest nascent wetland in the coastal zone of China and provides important ecosystem services. However, the environment and biodiversity of the Yellow River Delta have recently been seriously threatened by the widespread invasion of *S. alterniflora*. In this study, we used the Illumina MiSeq sequencing to explore the shifts in soil bacterial and fungal diversity and community composition following *S. alterniflora* invasion in the Yellow River Delta to better understand the invasion mechanism of *S. alterniflora*. Specifically, our study aimed to address two scientific questions: (1) How do the community structure and co-occurrence networks of soil bacteria and fungi change following *S. alterniflora* invasion? And (2) What are the key factors driving changes in soil microbial community following *S. alterniflora* invasion in the estuarine wetland?

## 2 Materials and methods

### 2.1 Study area and soil sampling

The study area is located in the Yellow River Delta, Shandong Province, China (37°40′–38°10′N, 118°40′–119°20′E). The mean annual precipitation in this region is 530–630 mm, and the mean annual temperature is 11.5–12.4°C. The invasive plant *S. alterniflora* was initially introduced to the Yellow River Delta in 1990 for coastal restoration, and its distribution area remained relatively stable until 2010. However, after 2010, *S. alterniflora* began to expand exponentially (Liu et al., 2018). By 2020, it had exceeded 5,000 ha and spread throughout the intertidal zone of the Yellow River Delta (Li H. et al., 2022).

In June 2022, we selected five representative sites where *S. alterniflora* had invaded. Bulk soil was collected from the five invaded sites and three adjacent sites covered by *Suaeda salsa* (Supplementary Figure S1, *S. alterniflora* and *S. salsa* coexisted on the sites 1–3), as *S. salsa* is the native species competing with *S. alterniflora* in this region. Specifically, soil samples were collected from the top 0–20 cm layer after visible plant residues were removed from the soil surface. Three replicates were collected at each site, and each replicate consisted of a mixture of four soil samples. The distance between different replicates was greater than 50 m. The collected soil samples were then divided into two portions. One portion was stored on dry ice and shipped as soon as possible to be stored in a −80°C refrigerator before soil microbial sequencing. The other portion of the samples was stored at 4°C and then used for soil physicochemical analysis.

### 2.2 Soil physicochemical analysis

Soil pH and electrical conductivity (EC) were measured using a pH meter (Leici PHS-3E) and an EC meter (Leici DDS-307A), respectively, with a soil: water ratio of 1:5. Soil organic carbon (SOC) was quantified using the potassium dichromate oxidation method with external heating. Total nitrogen (TN) and total phosphorus (TP) content in soil were measured using the Kjeldahl digestion method and the H_2_SO_4_-HClO_4_ digestion colorimetric method, respectively. Soil ammonium nitrogen (NH_4_^+^-N), nitrate nitrogen (NO_3_^−^-N), and available phosphorus (AP) were measured through a spectrophotometric method. Soil salinity was determined using the sand bath method.

### 2.3 Microbial DNA extraction, PCR amplification, and Illumina sequencing

Microbial DNA extraction from 0.5 g soil samples was carried out following the manufacturer’s instructions using the E.Z.N.A.^®^ soil DNA kit (Omega Bio-Tek, Norcross, GA, United States). After the extraction, the DNA integrity was verified using 1% agarose gel electrophoresis, and the DNA concentration and purity were measured using the NanoDrop 2000 spectrophotometer (Thermo Scientific, Wilmington, DE, United States). The extracted DNA was used as a template for PCR amplification targeting the V3–V4 hypervariable region of the bacterial 16S rRNA gene, employing the primer sequences 338F (5′-ACTCCTACGGGAGGCAGCAG-3′) and 806R (5′-GGACTACHVGGGTWTCTAAT-3′) (Yu et al., 2005). For the fungal ITS region, PCR amplification was conducted with the primer sequences ITS1F (5′-CTTGGTCATTTAGAGGAAGTAA-3′) and ITS2R (5′-GCTGCGTTCTTCATCGATGC-3′) (Tedersoo et al., 2015). The PCR amplification protocol comprised an initial denaturation step at 95°C for 3 min, followed by denaturation at 95°C for 30 s, annealing at 55°C for 30 s, and extension at 72°C for 30 s, repeated for 25 cycles, concluding with a final extension at 72°C for 10 min. Three replicates were performed under identical experimental conditions. Each template DNA was amplified three times, and the PCR products were pooled together after 2% agarose gel electrophoresis and recovered using the AxyPrep DNA Gel Recovery Kit (Axygen Biosciences, Union City, CA, United States). The recovered PCR products were eluted with Tris-HCl buffer and then subjected to 2% agarose gel electrophoresis. Based on preliminary quantification results from gel electrophoresis, the PCR products were quantified using the Quantus^™^ Fluorometer (Promega, United States). The products were then mixed in the corresponding proportions for Miseq library construction and sequenced on the Illumina MiSeq platform according to the sequencing requirements.

### 2.4 Bioinformatic analyses

The fastp (v0.19.6) and Flash (v1.2.7) program were used to combine and quality-check the raw 16S rRNA sequences. Raw sequences with average quality score <20 or a read lengths of <50 bp were filtered, and the mismatch ratio of the spliced sequences should not exceed 0.2. ASVs (amplicon sequence variants) were generated after sequence denoising using DADA2 software. The taxonomic annotation of each sequence was performed using the RDP classifier (v11.5) by aligning against the Silva 16S rRNA database (v138) with a similarity threshold set at 70%. QIIME2 was used to classify species and assess community diversity. Additionally, to assess variations in functional groups, the functional composition of soil bacteria and fungi was predicted using FAPROTAX (Louca et al., 2016) and FUNGuild (Nguyen et al., 2016), respectively.

### 2.5 Data analysis

One-way analysis of variance (ANOVA) was performed to evaluate the effect of *S. alterniflora* invasion on soil physicochemical variables and microbial alpha diversity. Duncan’s multiple comparison test (*p* < 0.05) was used to explore the differences between invaded and natural sites for each parameter. Soil microbial alpha diversity was characterized using Hill’s Diversity index, which usually includes species richness, Shannon entropy index, and inverse Simpson index (Jost, 2006). Changes in soil microbial beta diversity were analyzed using non-metric multidimensional scaling (NMDS) based on the Bray–Curtis distance matrix. The differences in microbial community structure between invaded and natural sites were tested using similarity analysis (ANOSIM) and non-parametric multivariate analysis of variance (Adonis). The Bray–Curtis dissimilarity was used as an indicator of beta diversity, while the Wilcoxon rank-sum test was employed to determine the difference. The Wilcoxon rank-sum test was used to compare the differences across all soil microbial phyla and functional groups. In constructing the microbial co-occurrence network, ASVs that were present in all samples and ranked among the top 200 were retained. The network was built using a Spearman correlation coefficient |*r*| > 0.7 and a false discovery rate (FDR) correction with *p* < 0.05. In this network, each node represents an ASV, and each edge represents the correlation between nodes. Gephi software (0.9.7) was used to visualize the microbial network and calculate network-related attributes to evaluate its complexity, such as the number of nodes, links, and average path length. Cohesion was used to display the relationship among species, with positive cohesiveness indicating better ability to cooperate and negative cohesion indicating greater conflict among species. Natural connectivity (network stability) was calculated by randomly removing network nodes. Random forest modeling and heat maps were used to evaluate the relationships between soil physicochemical properties and microbial community diversity and the relative abundance of dominant phyla. Data processing and visualization were all performed using R (4.1.0).

## 3 Results

### 3.1 Soil physicochemical properties

Notable changes in soil NH_4_^+^-N, salt, and pH occurred after the invasion of *S. alterniflora* (Table 1). Soil NH_4_^+^-N content at the invaded sites was 8.05 ± 0.31 mg kg^−1^, which was significantly higher than that at the native sites (6.62 ± 0.27 mg kg^−1^) (*p* < 0.01). On the contrary, soil salt content significantly decreased from 13.05 ± 1.51 g kg^−1^ in the non-invaded areas to 8.8 ± 0.86 g kg^−1^ in the *S. alterniflora* invasion areas (*p* < 0.05). Soil pH also significantly decreased after *S. alterniflora* invasion (*p* < 0.05). Other soil characteristics, including soil NO_3_^−^-N, TP, SOC, and EC, did not show significant differences between the invaded and native sites (*p* > 0.05, Table 1).

**Table 1:** Soil physicochemical properties under invasive and native vegetation.

	SWC (%)	SOC (g kg^−1^)	TN (g kg^−1^)	TP (g kg^−1^)	NH_4_^+^-N (mg kg^−1^)	NO_3_^−^-N (mg kg^−1^)	AP (mg kg^−1^)	pH	Salt (g kg^−1^)	EC (ms cm^−1^)
*S. alterniflora*	0.27 ± 0.01 a	3.36 ± 0.6 a	0.54 ± 0.06 a	0.45 ± 0.04 a	8.05 ± 0.31 a	1.42 ± 0.14 a	35.49 ± 2.43 a	8.27 ± 0.08 b	8.8 ± 0.86 b	2.37 ± 0.21 a
*S. salsa*	0.25 ± 0.01 b	3.51 ± 0.47 a	0.63 ± 0.17 a	0.43 ± 0.07 a	6.62 ± 0.27 b	1.26 ± 0.06 a	35.63 ± 8.14 a	8.56 ± 0.04 a	13.05 ± 1.51 a	2.93 ± 0.17 a

SWC, soil water content; SOC, soil organic carbon; TN, total nitrogen; TP, total phosphorus; NH_4_^+^-N, ammonium nitrogen; NO_3_^−^-N, nitrate nitrogen; AP, available phosphorus; salt, salinity; EC, electrical conductivity.

### 3.2 Soil microbial alpha and beta diversity

The alpha diversity of soil bacterial and fungal communities exhibited different changes after the invasion of *S. alterniflora* (Table 2). For example, in the non-invaded areas, the Shannon entropy index of soil bacterial community was 829.65 ± 48.51, which decreased to 698.14 ± 28.29 after *S. alterniflora* invasion (*p* = 0.02). By contrast, the species richness and Shannon entropy index of soil fungal community did not change significantly following *S. alterniflora* invasion (*p* > 0.05).

**Table 2:** Soil microbial alpha diversity in native and invaded plots.

	Diversity index	*S. alterniflora*	*S. salsa*	*p*-value
Soil bacteria	Species richness	1234.27 ± 34.97	1699.56 ± 78.64	<0.001
	Shannon entropy	698.14 ± 28.29	829.65 ± 48.51	0.02
	Inverse Simpson	399.99 ± 29.36	343.89 ± 26.40	>0.05
Soil fungi	Species richness	380.73 ± 28.16	415.44 ± 64.22	>0.05
	Shannon entropy	56.05 ± 15.06	66.56 ± 24.13	>0.05
	Inverse Simpson	21.02 ± 7.37	23.29 ± 9.10	>0.05

**Figure 1:**
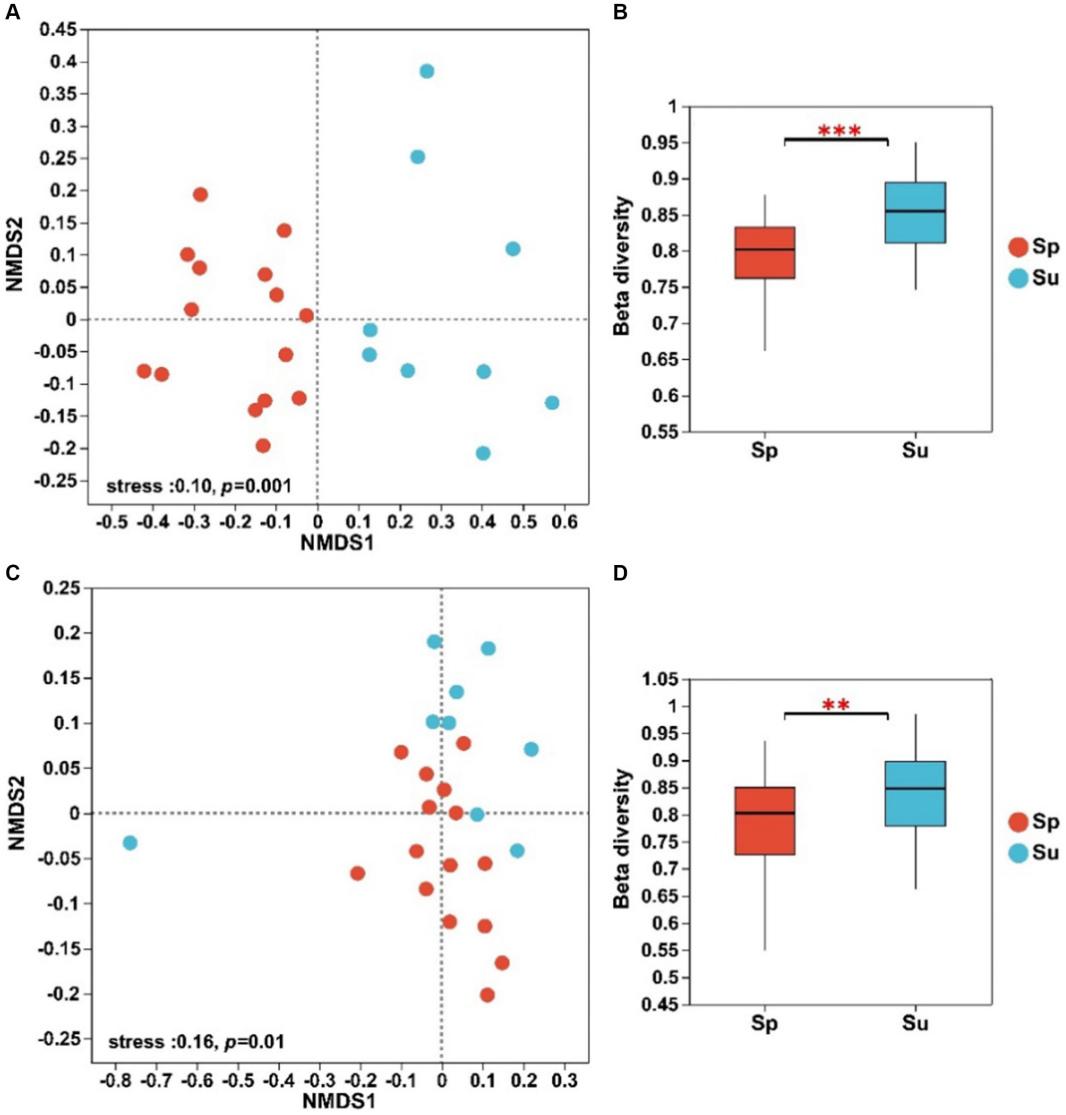
NMDS analysis and beta diversity of soil bacterial **(A,B)** and fungal **(C,D)** communities at the ASV level across all plots (^**^p < 0.01 and ^***^p < 0.001; *S. alterniflora*, i.e., Sp; *S. salsa*, i.e., Su).

The NMDS analysis showed significant differences in the structure of both soil bacterial and fungal communities between invaded and native sites (Figure 1). The ANOSIM and Adonis tests revealed that soil microbial beta diversity differed significantly across the invaded and native sites (Supplementary Table S1, *p* < 0.01). The Bray–Curtis dissimilarity showed that beta diversity of soil bacterial and fungal communities decreased with the invasion of *S. alterniflora* (Figures 1B,D; *p* < 0.01).

### 3.3 Soil microbial composition and functional groups

**Figure 2:**
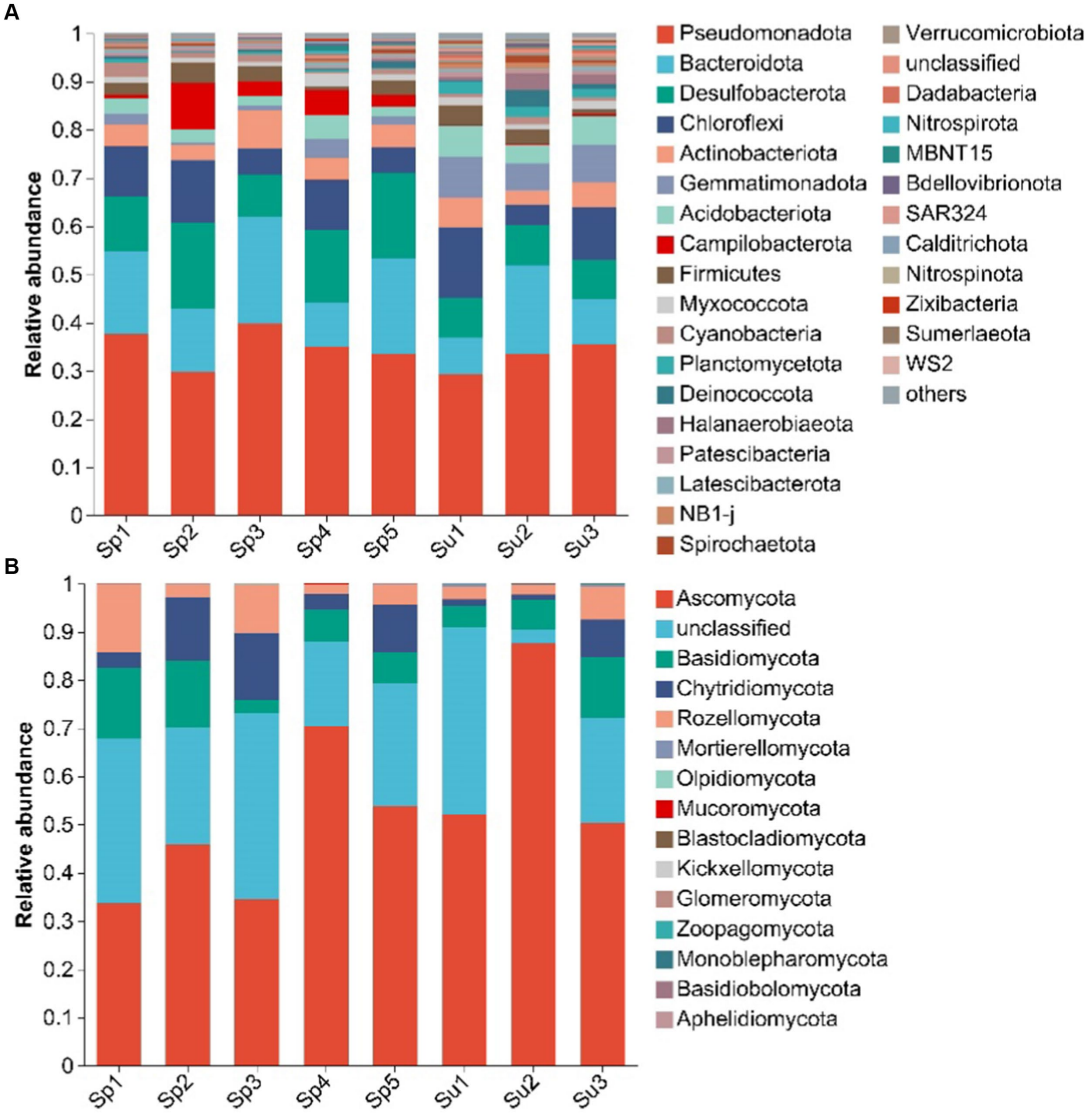
Dominant phyla of soil bacterial **(A)** and fungal **(B)** communities under invasive (*S. alterniflora*), and native vegetation (*S. salsa*). (Sp1–Sp5 represent the five sampling sites covered by *S. alterniflora*; Su1–Su3 represent the three sampling sites covered by *S. salsa*).

Pseudomonadota, Bacteroidota, and Desulfobacterota made up the majority of soil bacterial community, accounting for more than half of the overall abundance in this wetland (Figure 2A). The relative abundances of Bacteroidota (11.83–16.32%), Desulfobacterota (8.2–14.12%), and Campilobacterota (0.22–4.24%) significantly increased with plant invasion. In contrast, the relative abundance of Gemmatimonadota decreased from 7.33 to 1.87% after plant invasion (Supplementary Figure S2A, all *p* < 0.05).

Ascomycota, Basidiomycota, and Chytridiomycota were the main phyla of soil fungal community (Figure 2B). After the invasion of *S. alterniflora*, the relative abundance of Chytridiomycota (3.4–8.68%) significantly increased, whereas that of Mortierellomycota (0.38–0.07%) and Olpidiomycota (0.07–0.003%) significantly decreased (Supplementary Figure S2B, all *p* < 0.05).

According to FAPROTAX predictions, the relative abundances of soil bacterial functional groups associated with aerobic chemoheterotrophy, nitrate reduction, and nitrate respiration significantly increased after the invasion of *S. alterniflora* (*p* < 0.05). Conversely, functional groups associated with fermentation, hydrocarbon degradation, phototrophy, photoautotrophy, and photoheterotrophy significantly decreased (Supplementary Figure S3, all *p* < 0.05). Based on FUNGuild predictions, the relative abundance of pathotroph significantly decreased after *S. alterniflora* invasion, whereas the relative abundances of saprotroph and symbiotroph had no significant change (Supplementary Figure S4).

### 3.4 Soil microbial co-occurrence networks

**Figure 3:**
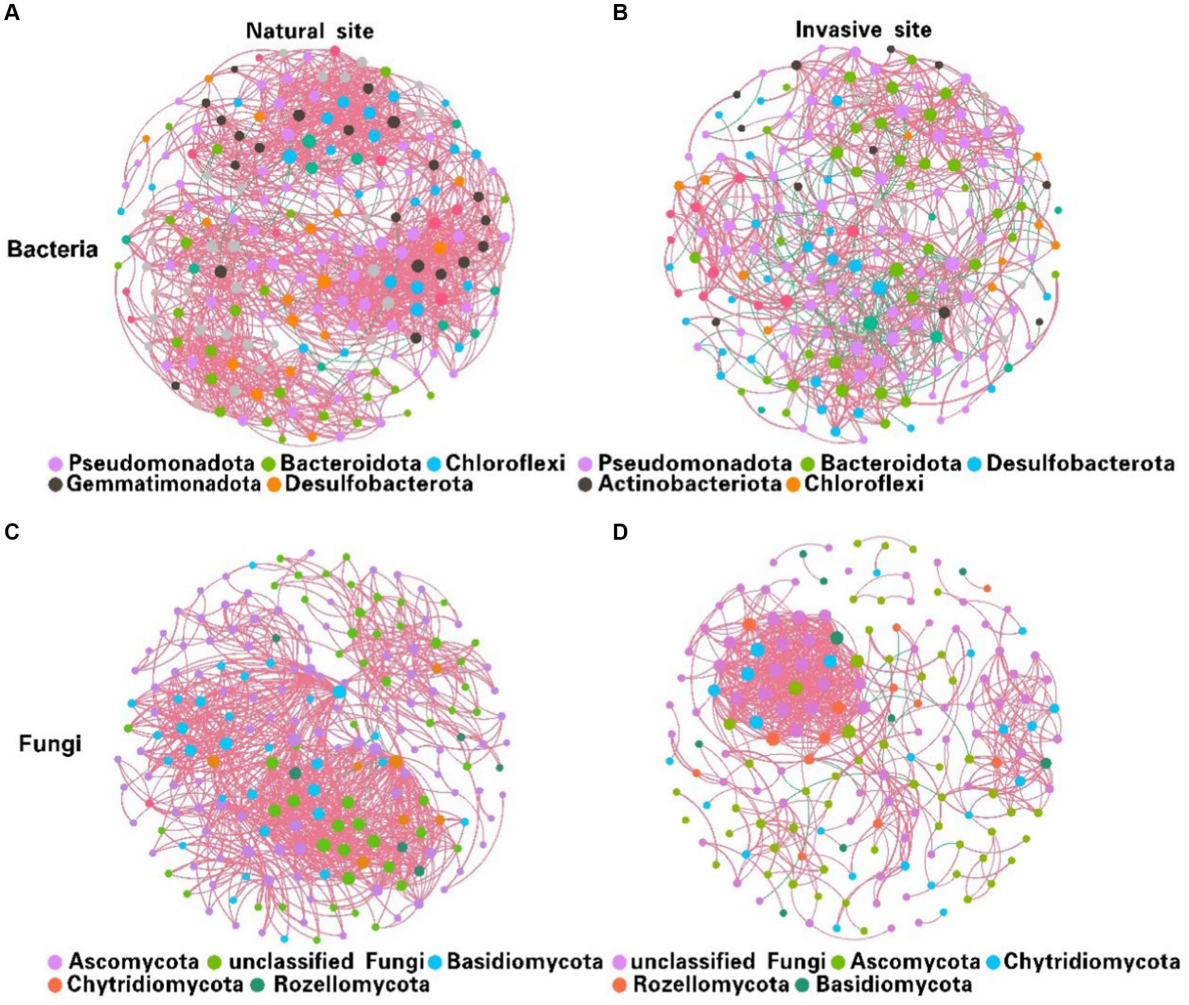
Variations in soil bacterial **(A,B)** and fungal **(C,D)** co-occurrence networks between natural **(A,C)** and invasive sites **(B,D)**. [Node size is proportional to its degree, and each line between pairs of nodes represents positive (pink) or negative (green) interactions, with line thickness indicating the magnitude of the Spearman coefficient].

**Figure 4:**
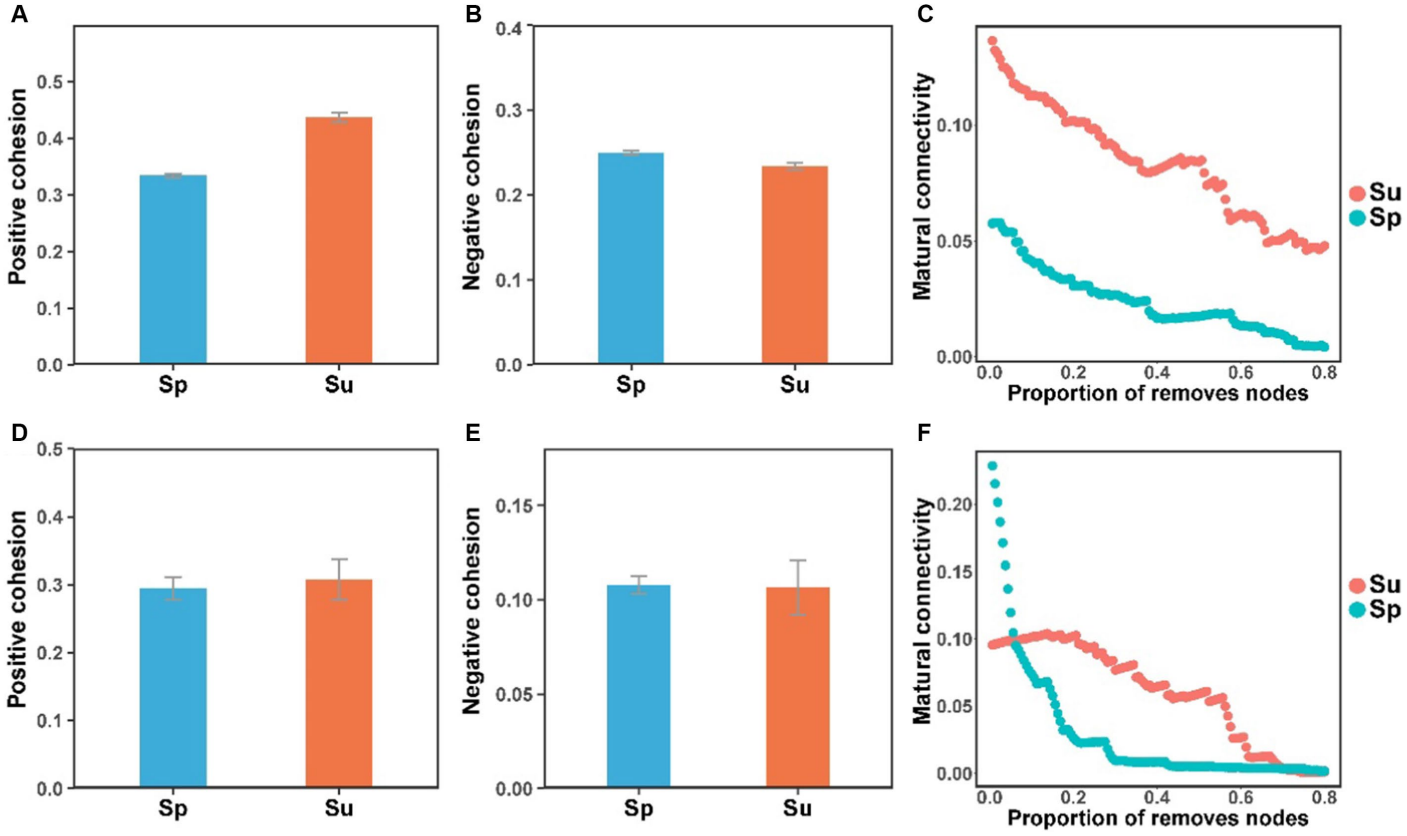
The cohesion and natural connectivity of soil bacterial **(A–C)** and fungal **(D–F)** communities at the natural and invasive sites (*S. alterniflora*, i.e., Sp; *S. salsa*, i.e., Su).

Co-occurrence network analysis suggested that the invasion of *S. alterniflora* simplified the complexity of the soil bacterial and fungal community networks (Figure 3). Specifically, the number of nodes in the networks of soil bacterial and fungal communities were higher in the non-invaded areas, with 197 nodes for bacteria and 198 nodes for fungi, in contrast to the invaded areas with 187 nodes for bacteria and 172 nodes for fungi. In invaded soil, the number of links in the microbial networks was lower than in native soil for both bacteria (806 vs. 1,524) and fungi (668 vs. 1,007). Additionally, the average degree of co-occurrence networks for soil bacteria and fungi decreased by 44 and 24%, respectively (Supplementary Tables S2, S3). Furthermore, soil bacterial and fungal communities at the natural sites had higher levels of positive cohesion and natural connectivity than those at the invasion sites, indicating a decreasing network stability and microbial inter-species cooperation after *S. alterniflora* invasion (Figure 4).

### 3.5 Relationship between soil microbial community and physicochemical properties

**Figure 5:**
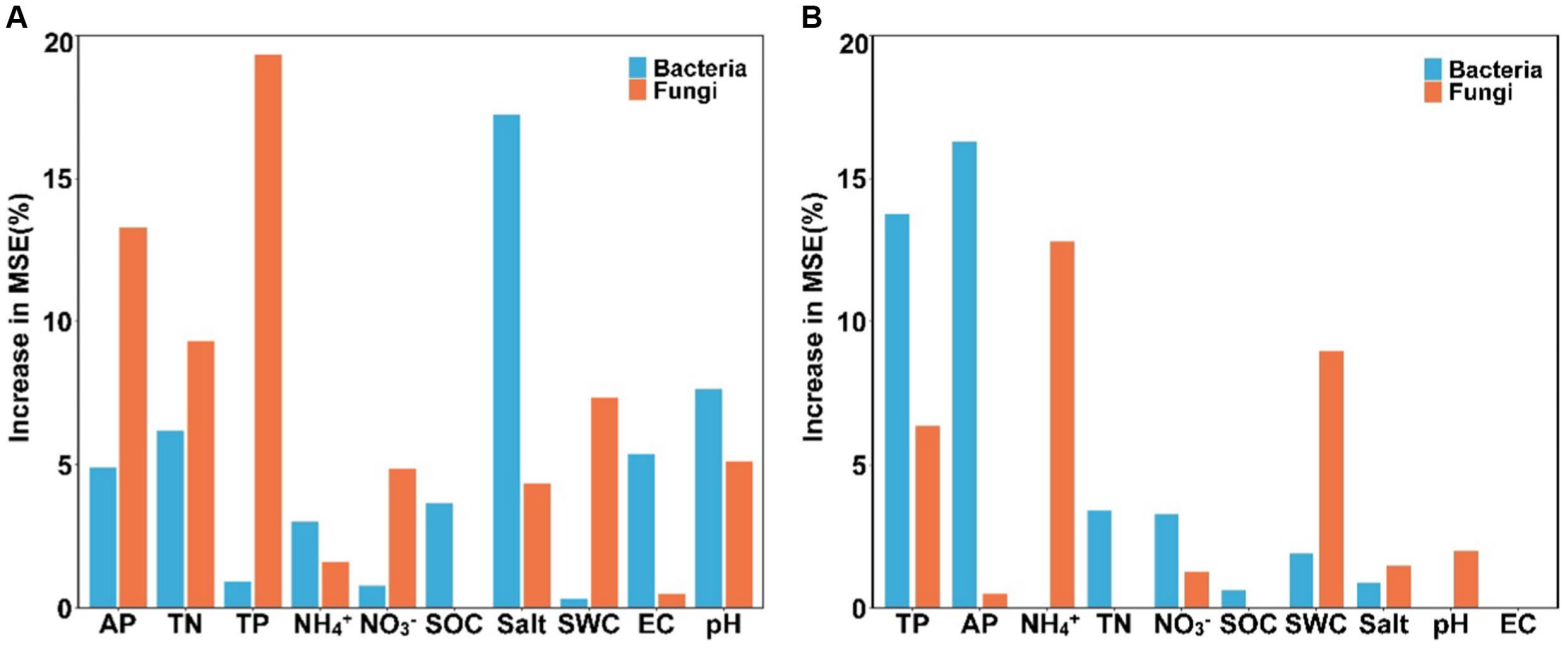
The main factors predicting the alpha **(A)** and beta **(B)** diversity of soil bacterial and fungal communities (SWC, soil water content; SOC, soil organic carbon; TN, total nitrogen; TP, total phosphorus; NH_4_^+^, ammonium nitrogen; NO_3_^−^, nitrate nitrogen; AP, available phosphorus; salt, salinity; EC, electrical conductivity).

**Figure 6:**
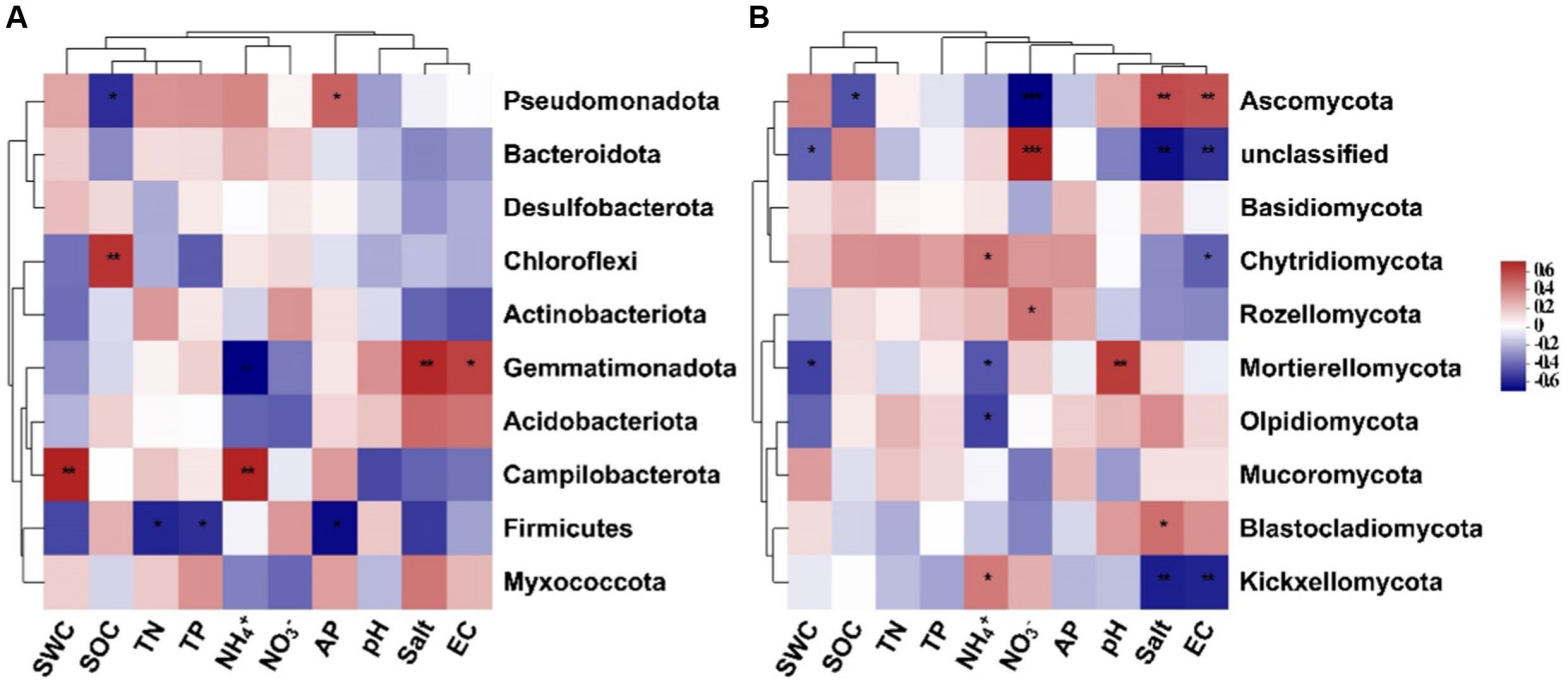
Spearman’s correlation between the top 10 dominant soil bacterial **(A)** and fungal **(B)** phyla and soil environmental variables (SWC, soil water content; SOC, soil organic carbon; TN, total nitrogen; TP, total phosphorus; NH_4_^+^, ammonium nitrogen; NO_3_^−^, nitrate nitrogen; AP, available phosphorus; salt, salinity; EC, electrical conductivity).

Results of the random forest analysis demonstrated that soil salinity content and pH were the primary factors affecting soil bacterial alpha diversity. In contrast, soil TP and AP contents played crucial roles in regulating soil fungal alpha diversity (Figure 5A). Soil bacterial beta diversity was mainly influenced by physicochemical attributes, particularly AP and TP, while soil fungal beta diversity was influenced primarily by NH_4_^+^-N and SWC levels (Figure 5B). The 10 dominant phyla of soil bacterial and fungal communities were significantly correlated with soil available nutrients and salinity content (Figure 6, *p* < 0.05). Overall, the diversity and composition of soil bacterial and fungal communities were largely determined by the interactions of various soil variables.

## 4 Discussion

### 4.1 *Spartina alterniflora* invasion alters soil microbial diversity and community composition

Higher microbial diversity has been recognized as an effective strategy for promoting the adaptability and successful invasion of alien plants (Zheng et al., 2019). However, our study revealed a significant decline in the α-diversity of soil bacterial community in *S. alterniflora* invaded areas (Table 2). This may be due to the duration of *S. alterniflora* invasion, with more longer years of invasion (>5 years) leading to decreasing soil bacterial α-diversity (Zhang G. et al., 2020). Moreover, a global meta-analysis showed that soil microbial α-diversity did not change significantly after plant invasion due to the varying native ecosystem types and physiological characteristics of invasive plant species (Torres et al., 2021). Contrasting the bacterial community, the α-diversity of soil fungi showed no significant change after *S. alterniflora* invasion. This discrepancy could be attributed to the different sensitivities of soil bacteria and soil fungi to environmental changes. For instance, one study showed that long-term nitrogen inputs induced changes in the α-diversity of soil bacteria in semi-arid grasslands without significant changes in soil fungi (Liu et al., 2021). In summary, our findings indicate that soil bacterial communities may be more sensitive to *S. alterniflora* invasion than fungal communities in the coastal wetlands.

The shifts observed in soil bacterial and fungal composition suggest that different microbial groups respond differently to *S. alterniflora* invasion. Previous research has found that the relative abundance of the Bacteroidota phylum generally increases in soils with *S. alterniflora* invasion, while Gemmatimonadota and Acidobacteriota display contrasting trends (Zhang G. et al., 2020). Similarly, our results revealed that *S. alterniflora* invasion increased the relative abundances of Bacteroidota, Desulfobacterota, and Campilobacterota within the soil bacterial community, while decreasing the relative abundances of Gemmatimonadota and Acidobacteriota (Supplementary Figure S2A). Acidobacteriota and Gemmatimonadota are generally considered to be slow-growing oligotrophic bacterial groups adapted to resource-limited soil environments (Verzeaux et al., 2016). In contrast, Bacteroidota is widely present in coastal wetlands and is typically regarded as copiotrophic bacteria, preferring nutrient-rich environments (Yang et al., 2020). Therefore, with the increase in soil nutrient resources following *S. alterniflora* invasion, copiotrophic bacterial groups may become more dominant. Additionally, our study revealed a rise in the relative abundance of Desulfobacterota following *S. alterniflora* invasion (Supplementary Figure S2A), which is consistent with another study that reported a noticeable rise in the quantity of sulfate-reducing bacteria in soils following *S. alterniflora* invasion in salt marshes (Zhang G. et al., 2020). Sulfate-reducing bacteria is the main species in the phylum Desulfobacterota, which can produce harmful substances such as hydrogen sulfide and reduce the available iron content in the soil. These actions can directly or indirectly harm other vegetation in invaded areas (Zeleke et al., 2013). Therefore, the increase in the relative abundance of Desulfobacterota may offer favorable conditions for the expansion of *S. alterniflora*. For soil fungal community, *S. alterniflora* invasion significantly increased the relative abundance of Chytridiomycota (Supplementary Figure S2B). This fungal phylum specializes in the degradation of complex organic compounds, such as cellulose and chitin (Song et al., 2019). This suggests that the invasion of *S. alterniflora* may induce extensive decomposition of recalcitrant organic matter in wetland soils, consequently providing more nutrient sources for the growth of *S. alterniflora*.

### 4.2 *Spartina alterniflora* invasion affects soil microbial functions and simplifies their co-occurrence networks

Soil microbial functional groups participate in the biogeochemical cycles and are closely related to soil nutrient status and plant growth (Wu et al., 2016; Liu et al., 2020). Our results indicate that the abundances of various functional microbial groups in soil bacterial community change after the invasion of *S. alterniflora*, which may contribute to the success of its invasion (Supplementary Figure S3). For example, aerobic chemoheterotrophic bacteria and nitrate-reducing bacteria significantly increased under the invasion of *S. alterniflora*. The enrichment of aerobic chemoheterotrophic and chemolithotrophic bacteria in soils can significantly enhance the rate of organic matter oxidation, assisting in more efficient carbon sources (Zhang et al., 2018). Nitrate-reducing bacteria can convert nitrogen into readily absorbable ammonium through nitrate dissimilatory reduction metabolism, thus improving the plant’s efficiency in absorbing and utilizing nitrogen resources (Pandey et al., 2019). As for soil fungi, our study found a significant reduction in the relative abundance of pathogenic fungi (Supplementary Figure S4). This change may be attributed to the large amount of *S. alterniflora* litter and its rapid decomposition rate, which greatly stimulates nutrient cycling in invaded areas, thereby increasing the proportion of saprotrophic fungi and reducing the proportion of pathogenic fungi. In summary, *S. alterniflora* invasion has caused a transformation in the composition and function of soil microbial communities, with a pronounced impact on specific microbial functional groups engaged in organic matter degradation. This could accelerate soil organic matter decomposition and affect the balance of soil nutrient cycling.

Soil microorganisms can form positive or negative, direct or indirect ecological connections through interactions such as competition, facilitation, and inhibition (Faust et al., 2012). Co-occurrence network analysis provides a standardized framework to study the relationships among different microbial groups, helping us better understand their interactions and their impacts on the environment and hosts (Wagg et al., 2019). In this study, the number of nodes, links, and the average degree of soil microbial network declined dramatically with the invasion of *S. alterniflora* (Supplementary Tables S2, S3). This indicates that *S. alterniflora*’s invasion simplifies the microbial co-occurrence network and weakens soil microbial interactions. These findings are inconsistent with recent research that found an increased complexity in the soil bacterial co-occurrence network following *S. alterniflora* invasion (Zhang G. et al., 2020). Conversely, another study exhibited diminished interactions among soil fungal communities after the invasion of *S. alterniflora* in salt marshes (Zhang et al., 2021). The inconsistent results may be related to soil microbial species, nutrient resource availability, and microbial diversity. For example, changes in nutrient inputs consequent to plant invasion can significantly affect the interactions among different microbial communities (Neutel et al., 2007). Low-diversity communities could reduce the network complexity because they are generally composed of similar species competing for limited resources, which may weaken microbial connectivity (Zhang et al., 2021). Our research revealed that *S. alterniflora* invasion decreased the α-and β-diversity of soil microbial communities while concurrently reducing the complexity of microbial networks. Moreover, the lower soil microbial natural connectivity in invaded areas suggests a decline in the stability of soil microbial communities. In short, *S. alterniflora* invasion weakens the interrelationships among soil microbial species and may further impair their ability to provide ecosystem services.

### 4.3 Soil physicochemical properties combine to drive changes in soil microbial diversity and structure

The expansion of invasive plants can significantly alter the nutrient dynamics and soil physicochemical properties (Tamura and Tharayil, 2014; Yuan et al., 2014). Our study found that *S. alterniflora* invasion dramatically changed the basic soil physicochemical parameters, as shown by the increase in soil ammonium nitrogen concentration and decrease in soil salinity and pH (Table 1). The decrease in soil pH caused by *S. alterniflora* invasion differs from the findings of a previous study. In the Minjiang River estuary wetland in China, the invasion of *S. alterniflora* significantly increased soil pH (Gao et al., 2017). The divergence may be attributed to various factors, such as differences in soil type and the invasion duration. In addition, our study found a decrease in soil salinity in areas invaded by *S. alterniflora*. This phenomenon may be related to the salt excretion traits of *S. alterniflora*, which can partially reduce soil salinity (Tang et al., 2014).

Changes in soil environmental factors, such as soil nutrients, pH, and salinity, can directly or indirectly influence the diversity and abundance of soil microbial communities. In our study, soil nutrient levels, such as soil available phosphorus and nitrogen contents, drove the changes in soil microbial diversity and structure. This observation underscores that *S. alterniflora* invasion can alter soil nutrient status and thus change microbial community structure, which confirms the pivotal role of soil nutrient availability in driving the succession of soil microbial communities within terrestrial ecosystems (Yang et al., 2019). Like soil nutrients, other soil physicochemical parameters, such as soil salinity content and pH, have a profound effect on soil bacterial alpha diversity (Figure 5). Soil salinity and EC also affect the relative abundance of some soil bacterial and fungal groups (Figure 6). Previous research has shown that soil salinity is a key driving factor in shaping the diversity and composition of soil microbial communities in the Yellow River Delta (Zhang et al., 2021). Consistently, our findings demonstrate significant correlations between soil salinity and various bacterial and fungal groups, such as Gemmatimonadota, Ascomycota, and Kickxellomycota. Besides, soil pH can affect plant nutrient accumulation, modify soil microenvironment, and subsequently alter soil microbial communities (Mamet et al., 2017). Thus, environmental variables during plant invasion may interact with each other, resulting in differential shifts in soil microbial structure. Further, our findings are based on a single sampling event, which may be a limitation to detect the effects of dynamic soil available nutrients and salinity on soil microbial communities in this area. Future research should increase the frequency of soil sampling to more comprehensively examine changes in soil microbial communities and their underlying drivers following *S. alterniflora* invasion.

## 5 Conclusion

This study investigated the changes in soil bacterial and fungal communities in the Yellow River Delta Wetland following *S. alterniflora* invasion. *S. alterniflora* invasion considerably decreased soil bacterial α-diversity but did not significantly change soil fungal diversity. Furthermore, *S. alterniflora* invasion significantly altered the community composition of soil microorganisms. The relative abundances of functional groups involved in carbon and nitrogen cycling in the soil bacterial community increased. Dramatically, *S. alterniflora* invasion weakened the interactions among differential soil microbial species and reduced soil microbial network stability. These changes in soil bacterial and fungal communities were primarily controlled by soil available nutrients and soil salinity. This study provides new insights into the functional and symbiotic dynamics of soil bacterial and fungal communities following *S. alterniflora* invasion.

## Data availability statement

The datasets presented in this study can be found in online repositories. The names of the repository/repositories and accession number(s) can be found at: https://www.ncbi.nlm.nih.gov/, PRJNA1021770; https://www.ncbi.nlm.nih.gov/, PRJNA1021774.

## Author contributions

TZ: Data curation, Formal analysis, Investigation, Writing – original draft. BS: Conceptualization, Funding acquisition, Supervision, Writing – original draft, Writing – review & editing. LW: Investigation, Visualization, Writing – original draft. YL: Supervision, Validation, Writing – original draft. YW: Project administration, Supervision, Writing – review & editing. MY: Validation, Writing – review & editing.
