# Supplementary material for: Spartina alterniflora invasion reduces soil microbial diversity and weakens soil microbial inter-species relationships in coastal wetlands
Source: Front Microbiol. 2024 Jul 31;15:1422534. doi: 10.3389/fmicb.2024.1422534 (PMC11325588; doi:10.3389/fmicb.2024.1422534)
Supplement: Supplementary file 1 [file Data_Sheet_1.pdf]

***Spartina alterniflora* invasion reduces soil microbial diversity and weakens soil microbial inter-species relationships in coastal wetlands**

Tao Zhang [1, 2, †], Bing Song [1, †, *], Luwen Wang [1], Yong Li [3], Yi Wang [1], Min Yuan [4]

[1] School of Resources and Environmental Engineering, Ludong University, Yantai 264025, China

[2] State Environmental Protection Key Laboratory of Wetland Ecology and Vegetation Restoration, Institute for Peat and Mire Research, Northeast Normal University, Changchun 130024, China

[3] Beijing Key Laboratory of Wetland Services and Restoration, Institute of Wetland Research, Chinese Academy of Forestry, Beijing 100091, China

[4] Department of Renewable Resources, University of Alberta, Edmonton T6G 2E3, Canada

[†] These authors contributed equally to this work and share first authorship.

* Correspondence to: Bing Song (E-mail address: songbing@ldu.edu.cn)

Fig. S1. Maps of the study location and sampling sites.

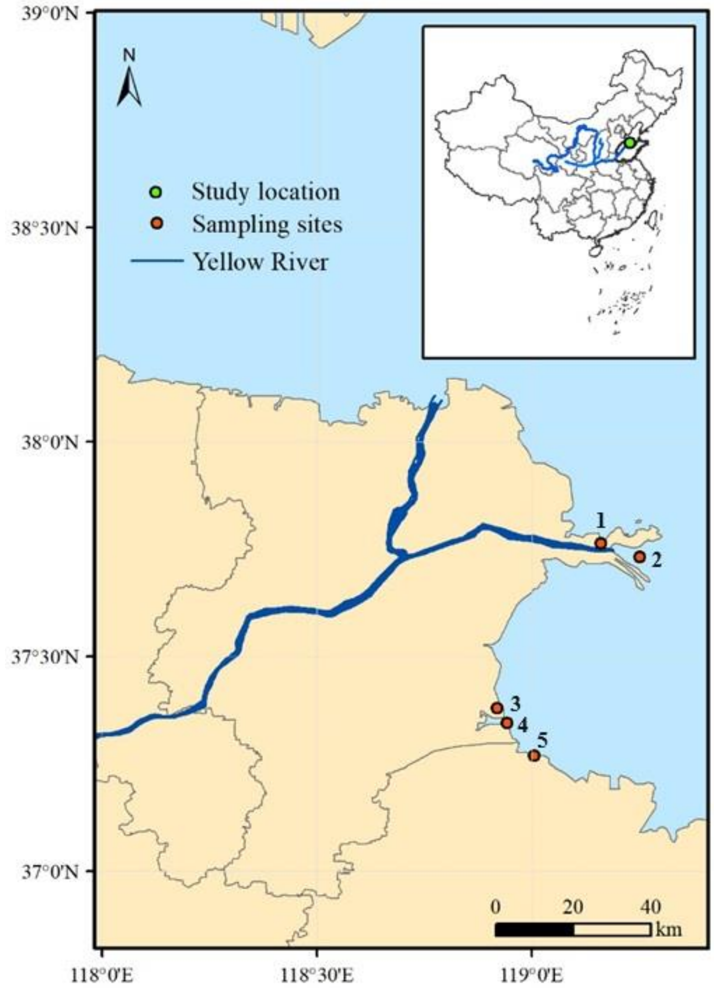

Fig. S2. Differences in the composition of soil bacterial (a) and fungal (b) communities between *S. alterniflora* (Sp) and *S. salsa* (Su) (Significant differences: *, $p<0.05$; **, $p<0.01$; ***, $p<0.001$)

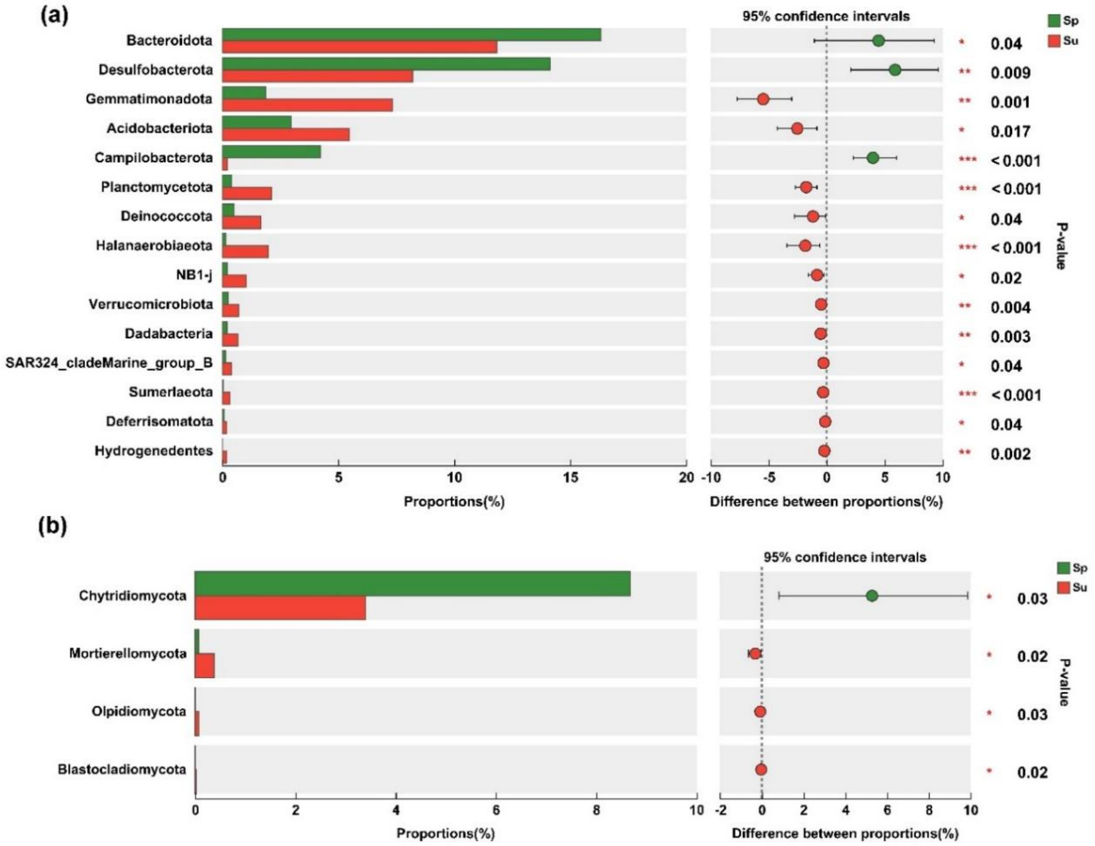

Fig. S3. Predicted changes in the relative abundances of soil bacterial functional groups based on FAPROTAX. (Sp: *S. alterniflora*; Su: *S. salsa*; significant differences: *, p<0.05; **, p<0.01; ***, p<0.001)

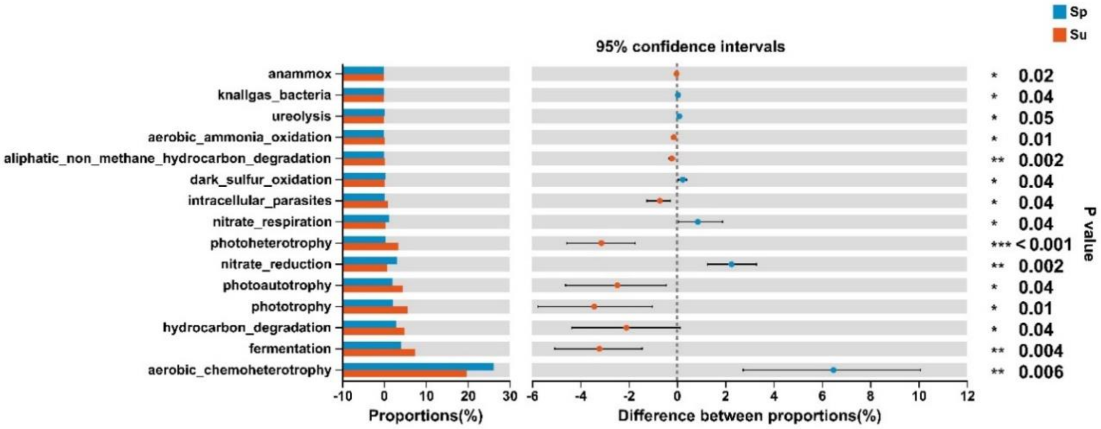

Fig. S4. Predicted changes in the relative abundances of soil fungal functional groups based on FUNGuild. (Sp: *S. alterniflora*; Su: *S. salsa*; *, p<0.05)

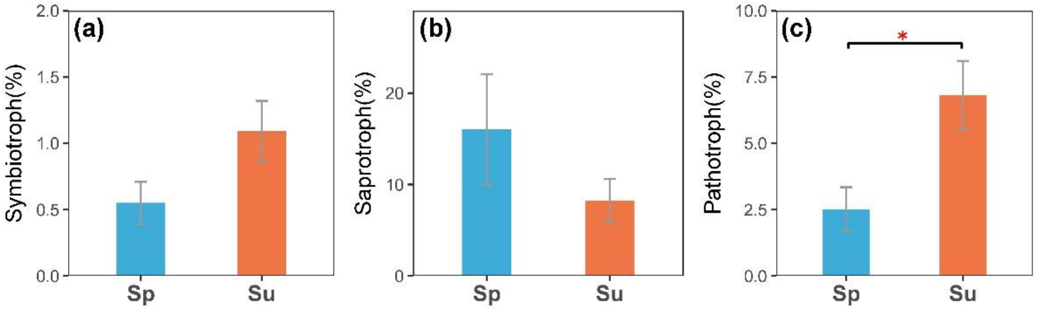

Table S1. Significance test of the effects of *S. alterniflora* invasion on soil microbial community based on Bray-Curtis dissimilarity matrix.

	ANOSIM		Adonis	
	R	*p*	F	*p*
Soil bacteria	0.78	0.001	4.46	0.001
Soil fungi	0.25	0.009	1.88	0.01

Table S2. The co-occurrence network properties of soil bacterial communities.

	Node	Link			Average degree	Modularity	Average clustering coefficient	Average path length
		Total	Positive	Negative				
S. alterniflora	187	806	80.02%	19.98%	8.62	0.71	0.43	3.58
S. salsa	197	1524	98.95%	1.05%	15.47	0.61	0.51	3.13

Table S3. The co-occurrence network properties of soil fungal communities.

	Node	Link			Average degree	Modularity	Average clustering coefficient	Average path length
		Total	Positive	Negative				
S. alterniflora	172	668	98.5%	1.5%	7.77	0.67	0.44	4.77
S. salsa	198	1007	99.21%	0.79%	10.17	0.63	0.32	4.16
